# Longitudinal Fasting Blood Glucose Trends and Mortality Risk in Mice Differs From That of Non-Human Primates and Humans

**DOI:** 10.1093/geroni/igaa057.2625

**Published:** 2020-12-16

**Authors:** Rafael de Cabo, Dushani Palliyaguru, Eric Shiroma, John Nam, SLAM Investigators, Luigi Ferrucci

**Affiliations:** National Institute on Aging, Bethesda, Maryland, United States

## Abstract

Longitudinal studies in humans have led to the development of strong predictors of outcomes of health, disease and mortality. Translation from model organisms to human has been faced with species-specific regulation of metabolic function and challenged by the lack of longitudinal studies addressing trajectories of change that can be used, as in humans to predict outcomes. Here we compare longitudinal predictors of health and mortality of three major metabolic indices among mice, non-human primates and humans. Longitudinal fasting blood glucose, body weight and body composition over the lifespan were compared across species, mice, Rhesus monkeys and humans. Survival analysis was conducted to calculate the risk of death for subjects with highest and lowest quartiles of fasting blood glucose. We will present data highlighting species-specific mechanisms of glucose homeostasis over the lifespan and its association with mortality.

